# *PCD* Recognizes Outstanding Student Research: Kersten et al on Using a Standard Classification Scheme to Identify Small Food Stores That Offer Healthy Options

**DOI:** 10.5888/pcd9.120129

**Published:** 2012-07-12

**Authors:** Samuel F. Posner

This week, *Preventing Chronic Disease* (*PCD*) publishes “Small Food Stores and Availability of Nutritious Foods: A Comparison of Database and In-Store Measures, Northern California, 2009,” (1) winner of the journal’s 2012 Student Research Contest. The winning article is authored by Ellen Kersten, a doctoral student in the Department of Environmental Science, Policy, and Management at the University of California, Berkeley. Her primary advisor is Rachel Morello-Frosch, PhD, MPH.

**Podcast: Interview with Author Ellen Kersten**

Ellen Kersten, a University of California, Berkeley PhD candidate and this year’s winner of PCD’s 2012 student research contest, investigates the availability of nutritious foods in small food stores in six predominantly urban counties in Northern California. PCD interviewed Kersten about her research and asked her what she has planned after graduation. Listen now.

We received 45 submissions this year, which was more than 4 times the number of submissions as last year, the contest’s first. The strong response this year reaffirms the primary goal of the contest: to recognize outstanding student research and give students an opportunity to publish their work. The contest also represents *PCD*’s commitment to promoting an open exchange of information and knowledge among researchers, practitioners, policy makers, and others who strive to improve the health of the public. The increase in the number of submissions made the selection process especially challenging for the submissions review committee. 

The food environment has generated great interest, including defining food deserts and determining the effect of the availability of healthy foods on diet and obesity. Kersten and colleagues examined one of the fundamental tools for exploring the food environment: North American Industry Classification System (NAICS) codes. NAICS is “the standard used by federal statistical agencies in classifying business establishments for the purpose of collecting, analyzing, and publishing statistical data related to the US business economy” (2). These codes are often used in food environment research and practice to classify food outlets; the classifications are then used by researchers as a proxy measure of the availability of healthy (or not-so-healthy) foods. NAICS codes are often assumed to be sensitive and specific — that apples are being compared to apples, or in this case, that similarly coded stores have similar offerings and do not vary by external characteristics, such as square footage or number of employees. Kersten and her colleagues thoughtfully conducted a field investigation to assess whether this assumption represents reality.

They found that the NAICS code for small grocery stores represents a heterogeneous set of stores, not a single, homogeneous group. The types and numbers of nutritious food items offered by the stores in the study sample varied substantially, suggesting that NAICS codes may not be appropriate for epidemiologic studies, including studies that seek to identify food deserts or determine the association of store characteristics with behavioral and health outcomes. This study illustrates that data designed for one purpose can pose problems when used for another purpose; they should be used only after carefully considering their reliability and validity in the context in which they are being used. This valuable information will affect future research and program planning.

Ms Kersten’s article also reminds all of us who are dedicated to improving the health and well-being of the public that issues are sometimes more complex than expected, that items are not always what they appear to be, and that the validity of the basic measurements on which we commonly rely must be ensured. During the review process, the relevance of this study became clear, and the measurement issues raised and their effect on research were readily apparent. Equally important, however, was the observation from a reviewer who works in the field that the findings would fundamentally change the way programs and policies are designed and developed. If high-need areas are to be correctly identified and given top priority, Kersten and colleagues astutely note, incentive programs designed to increase the offerings of healthy foods may require more information on community context than is available from food store classifications derived from commercially available codes.

We congratulate Ms Kersten for her contributions to the field of public health; findings from her work have immediate implications for research, programs, and policy. Her work also accurately reflects the quality of submissions to the *PCD* Student Research Contest. The submissions review process consisted of multiple rounds. Each of the 45 submissions was reviewed by at least 2 reviewers. Semifinalists and finalists were reviewed by a panel of 5 editorial board members, including the founding editor of *PCD* and the editor in chief.

The final decision was difficult. Many submissions were well-researched, well-written, and worthy of publication. *PCD* will be publishing 6 additional student articles in the coming months. All of the students who submitted manuscripts are to be commended on their good work and their advisors thanked for their guidance. We recognize that mentors are influential in the development of the next generation of scientists. Senior researchers and practitioners including *PCD* staff have a responsibility to mentor students so that the field continues to advance and the health of the population is optimized. The call for submissions to the Third Annual *PCD* Student Research Contest will be issued in July, and the submission deadline will be in early January 2013.
